# Striatal Gα_olf_/cAMP Signal-Dependent Mechanism to Generate Levodopa-Induced Dyskinesia in Parkinson’s Disease

**DOI:** 10.3389/fncel.2017.00364

**Published:** 2017-11-21

**Authors:** Satoshi Goto

**Affiliations:** ^1^Department of Neurodegenerative Disorders Research, Institute of Biomedical Sciences, Graduate School of Medical Sciences, Tokushima University, Tokushima, Japan; ^2^Parkinson’s Disease and Dystonia Research Center, Tokushima University Hospital, Tokushima, Japan

**Keywords:** olfactory type G-protein α subunit, levodopa-induced dyskinesia, Parkinson’s disease, dopamine, striatum

## Abstract

The motor symptoms of Parkinson’s disease (PD) result from striatal dopamine (DA) deficiency due to a progressive degeneration of nigral dopaminergic cells. Although DA replacement therapy is the mainstay to treat parkinsonian symptoms, a long-term daily administration of levodopa often develops levodopa-induced dyskinesia (LID). LID is closely linked to the dysregulation of cyclic adenosine monophosphate (cAMP) signaling cascades in the medium spiny neurons (MSNs), the principal neurons of the striatum, which are roughly halved with striatonigral MSNs by striatopallidal MSNs. The olfactory type G-protein α subunit (Gα_olf_) represents an important regulator of the cAMP signal activities in the striatum, where it positively couples with D_1_-type dopamine receptor (D_1_R) and adenosine A_2A_ receptor (A_2A_R) to increase cAMP production in the MSNs. Notably, D_1_Rs are primarily expressed in striatonigral MSNs, whereas D_2_Rs and A_2A_Rs are expressed in striatopallidal MSNs. Based on the evidence obtained from parkinsonian mice, we hypothesized that in the DA-denervated striatum with D_1_R hypersensitivity, a *repeated* and *pulsatile* exposure to levodopa might cause a usage-induced degradation of Gα_olf_ proteins in striatal MSNs, resulting in increased and decreased levels of Gα_olf_ protein in the striatonigral and striatopallidal MSNs, respectively. As a principal cause for generating LID, this might lead to an increased responsiveness to levodopa exposure in both striatonigral and striatopallidal MSNs. Our hypothesis reinforces the long-standing concept that LID might result from the reduced activity of the striatopallidal pathway and has important clinical implications.

## Introduction

By transducing extracellular signals carried by neuromodulators, the cyclic adenosine monophosphate (cAMP) signaling plays a crucial role in the regulation of neuronal activities in the brain. Multiple guanine nucleotide-binding protein (G-protein)-coupled receptor (GPCR) cascades regulate the intracellular levels of cAMP, which activates its key effector protein kinase A. Seven-transmembrane domain receptors can transmit extracellular signals to the intracellular signaling cascades through the activation of heterotrimeric G-proteins, which are composed of the guanine nucleotide-binding Gα subunit and the dimeric βγ subunits (Pierce et al., 2002). Gα_s_ is the predominant stimulatory G-protein subunit in the brain. However, in the striatum, Gα_s_ is replaced by the olfactory type G protein α subunit (Gα_olf_), which is encoded by the *GNAL* gene (Jones and Reed, 1989). Cellular Gα_olf_/cAMP signaling pathway represents a principal regulator for the striatal functions in normal physiological processes and pathological conditions (Hervé, 2011). It is worth noting that mutations in the *GNAL* gene have been identified as a cause for generating dystonia (Fuchs et al., 2013; Pelosi et al., 2017), suggesting that the Gα_olf_ function might participate in the brain circuit involving motor control.

The motor symptoms of Parkinson’s disease (PD) are caused by striatal dopamine (DA) deficiency, predominantly in the putamen, resulting from a progressive degeneration of nigrostriatal DA-producing cells (Kish et al., 1988; Goto et al., 1989). Although the DA replacement therapy remains the mainstay to treat PD symptoms, long-term exposure to dopaminergic drugs, particularly to the DA precursor levodopa, eventually causes adverse effects such as motor fluctuations and levodopa-induced dyskinesia (LID; Jenner, 2008; Calabresi et al., 2010; Bastide et al., 2015). LID is a major cause of disability in patients with PD, and occurs in approximately 80% of patients after 5 years of treatment with a daily administration of levodopa (Obeso et al., 1989; Luquin et al., 1992; Rascol et al., 2000). Importantly, once LID has been primed (or established), its severity progressively increases despite even when the used dosage of dopaminergic drugs is not increased (Brotchie, 2005). LID is known to be closely linked to the altered function of the DA signaling pathways in the striatum (Brotchie, 2005; Jenner, 2008; Bastide et al., 2015; Calabresi et al., 2016). It has also been suggested that LID is associated with the hypersensitivity of striatal MSNs to DA receptor stimulation and with ongoing deregulation of corticostriatal inputs, which activate striatal glutamate receptors, such as *N*-methyl-D-aspartate (NMDA) receptors (Brotchie, 2005; Jenner, 2008; Bastide et al., 2015; Calabresi et al., 2016). In this hypothesis article, we primarily considered the levodopa-induced changes in cellular Gα_olf_ protein levels in the DA-denervated striatum as the key mechanism to increase the striatal responsiveness to DA receptor stimulation in LID.

## Gα_olf_ Regulates The Agonist-Induced cAMP Production in Striatal Cells

**Figure 1:**
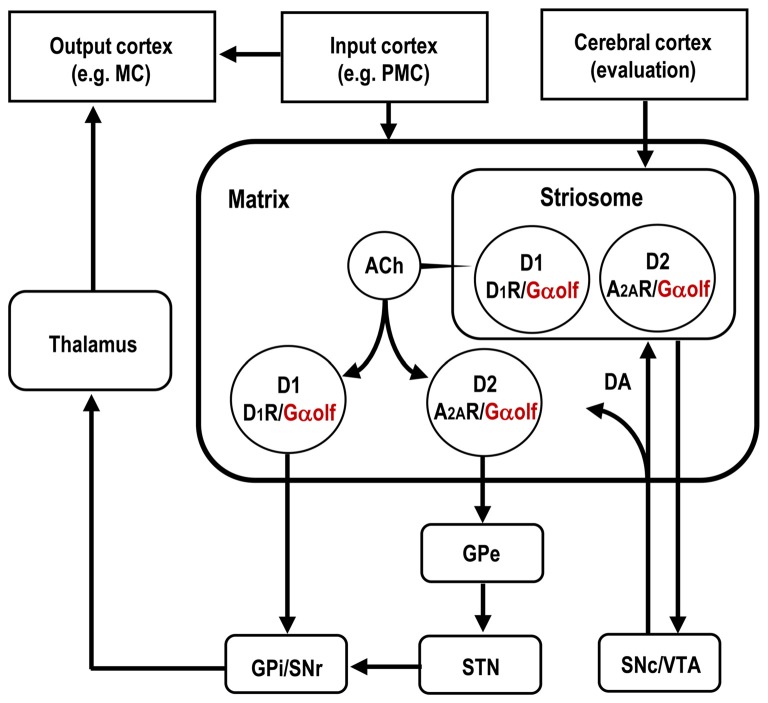
Distributional pattern of Gα_olf_ proteins in striatal medium spiny neurons (MSNs) that form the basal ganglia circuit. Gα_olf_ proteins are colocalized with DA D_1_ receptors (D_1_Rs) in the striatonigral MSNs (D1-cells), and with adenosine A_2A_ receptors (A_2A_Rs) in striatopallidal MSNs expressing DA D_2_ receptors (D_2_Rs; D2-cells). The striatonigral and striatopallidal pathways arising from the striosome are omitted in this scheme. ACh, acetylcholine; DA, dopamine; GPe, globus pallidus externa; GPi, globus pallidus interna; MC, motor cortex; PMC, premotor cortex; SNr, substantia nigra pars reticulata; SNc, substantia nigra pars compacta; STN, subthalamic nucleus; VTA, ventral tegmental area.

As being innervated by massive dopaminergic afferents originating from the midbrain, the striatum is highly enriched in DA receptors, which belong to a superfamily of GPCRs and are classified into two subtypes, D_1_- and D_2_-type receptors. Through their specific targeting of G proteins, the D_1_-type receptors (D_1_Rs) elicit the adenylyl cyclase type (AC) to increase the cAMP production, whereas the D_2_-type dopamine receptors (D_2_Rs) inhibit the cAMP production (Kebabian and Calne, 1979; Missale et al., 1998). Medium spiny neurons (MSNs) constitute more than 90% of the neuronal types in the striatum (Graybiel, 2008; Kreitzer, 2009; Gerfen and Surmeier, 2011). Anatomically, they are roughly halved with the MSN group to form the “direct” striatonigral pathway by the MSN group to from the “indirect” striatopallidal pathway (Crittenden and Graybiel, 2011; Gerfen and Surmeier, 2011; Calabresi et al., 2014). The striatonigral and striatopallidal MSNs express D_1_Rs and D_2_Rs, respectively. Moreover, the striatopallidal MSNs, but not the striatonigral MSNs, are enriched in adenosine A_2A_ receptors (A_2A_Rs), which are prototypical Gs-coupled receptors that elicit AC to increase cAMP production (Svenningsson et al., 1999; Schwarzschild et al., 2006; Fuxe et al., 2007). Figure 1 depicts the cell-type specific localization of Gα_olf_, D_1_R and A_2A_R among the striatal MSNs that constitute the basic circuits of the basal ganglia.

**Figure 2:**
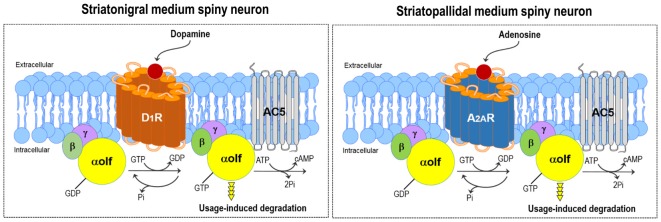
DA and adenosine-induced cAMP production in striatal MSNs. Gα_olf_ positively couples with the DA D_1_ receptor (D_1_R) and adenosine A_2A_ receptor (A_2A_R) to activate adenylyl cyclase type 5 (AC5) and subsequently increase cAMP production in striatonigral (*left*) and striatopallidal (*right*) MSNs, respectively. Thus, the DA-induced activation of D_1_R or adenosine-induced activation of A_2A_R leads to the degradation of the Gα_olf_ protein through the usage-dependent mechanism in striatonigral or striatopallidal MSN, respectively.

Gα_olf_ is highly expressed in all striatal MSNs including those expressing the D_1_Rs and A_2A_Rs (Kull et al., 2000; Hervé, 2011; Morigaki et al., 2017; see Figure 2). As Gα_olf_ positively couples with D_1_R and A_2A_R to activate the AC type 5 (AC5) and, thereby, increase the intracellular cAMP levels, it serves as the rate-limiting factor for both the D_1_R- and A_2A_R-dependent cAMP production in striatal MSNs (Kull et al., 2000; Corvol et al., 2001). The Gα_olf_ protein level plays a key role in regulating the D_1_R/cAMP- and A_2A_R/cAMP-signal activities of striatonigral and striatopallidal MSNs, respectively. The D_1_R/Gα_olf_-mediated increases in the cAMP levels cause the activation of the striatonigral MSNs (Hervé, 2011). On one hand, as D_2_R activation inhibits AC5 through G_i/o_ proteins but A_2A_R activation elicits AC5 through G_s/olf_ proteins (Kull et al., 2000), the A_2A_R/Gα_olf_-signal stimulation functionally opposes the actions of D_2_Rs on the striatopallidal MSNs (Schwarzschild et al., 2006; Fuxe et al., 2007).

## Subdivisional and Compartmental Localization of Gα_olf_ in The Striatum

Quantitative immunohistochemistry (IHC) has shown that the Gα_olf_ protein is unevenly distributed within the mouse striatum, where it is highly concentrated in the dorsolateral striatum (Morigaki et al., 2017). Since the dorsolateral portion of the mouse striatum corresponds to the motor territory in rodents and is analogous to the putamen in primates (Graybiel, 2008), this strategic expression of Gα_olf_ protein indicates that Gα_olf_ may function as the stimulatory G protein that has a tight link to the basal ganglia “motor” circuit (Alexander and Crutcher, 1990) at the striatal level. With respect to the striatal compartments, there was a differential localization of Gα_olf_ with higher densities of Gα_olf_ proteins in the striosomes relative to the matrix compartment (Sako et al., 2010; Ruiz-DeDiego et al., 2015; Morigaki et al., 2017). This suggests that Gα_olf_ may be a key molecule that determines differential responses between the striosome and matrix compartments to the D_1_R or A_2A_R activation in the striatum at maturity.

## Homeostatic Regulation of The Cellular Gα_olf_ Protein Levels in The Striatum

Rodent animal models for PD (Iderberg et al., 2012; Francardo and Cenci, 2014) have so far been used to elucidate the regulatory mechanism for the striatal expression of Gα_olf_. In line with the evidence that there is a significant increase in Gα_olf_ protein levels in the putamen of patients with PD (Corvol et al., 2004), a dramatic increase in Gα_olf_ protein levels has been identified in the DA-depleted striatum of rats (Hervé et al., 1993; Marcotte et al., 1994; Penit-Soria et al., 1997; Corvol et al., 2004; Rangel-Barajas et al., 2011) and mice (Alcacer et al., 2012; Ruiz-DeDiego et al., 2015; Morigaki et al., 2017) with nigrostriatal 6-hydroxydopamine lesions. However, this upregulation of the Gα_olf_ protein levels is not associated with a parallel increase of the Gα_olf_ mRNA expression. Accordingly, the homeostatic regulation of Gα_olf_ protein levels is thought to occur through post-translational mechanisms in the striatum, where the altered expression of the Gα_olf_ protein depends directly on its usage rate (Hervé, 2011). The persistent lack in the use of D_1_R and Gα_olf_ could lower the Gα_olf_ degradation rate and thereby result in the accumulation of Gα_olf_ protein in the DA-denervated striatum of PD models. In agreement with this hypothesis, a total lack of D_1_Rs by D_1_R gene targeting induces a significant increase of the Gα_olf_ protein levels without any changed expression of Gα_olf_ mRNAs in the striatum of mutant mice (Hervé et al., 2001). In contrast, the decreased levels of striatal Gα_olf_ proteins were found in mutant mice lacking the DA transporter (Hervé et al., 2001), which exhibit a marked increase in the extracellular DA levels leading to persistent activation of D_1_Rs in the striatum (Giros et al., 1996). Importantly, the lack of A_2A_Rs in homozygous A_2A_R knock-out mice (Ledent et al., 1997) also results in an upregulation of Gα_olf_ proteins with no obvious changes in the levels of Gα_olf_ transcripts (Hervé et al., 2001). Collectively, the agonist-induced activation of D_1_Rs (Hervé et al., 2001; Corvol et al., 2004, 2007; Alcacer et al., 2012; Ruiz-DeDiego et al., 2015) or A_2A_Rs (Hervé et al., 2001) might lead to the degradation of Gα_olf_ proteins in striatal MSNs through posttranslational usage-dependent mechanism (see Figure 2).

## Gα_olf_ Protein Levels in Striatonigral and Striatopallidal MSNs in LID

On the hypothesis that the upregulation of the Gα_olf_ protein levels results from the disuse of the D_1_Rs in the DA-depleted striatum in rodent models for PD, several studies with IHC and western blot analyses revealed that the Gα_olf_ could be returned to normal levels by DA replacement with a daily exposure to levodopa in rodent models for PD with LID (Corvol et al., 2004; Rangel-Barajas et al., 2011; Ruiz-DeDiego et al., 2015; Morigaki et al., 2017). With respect to the striosome-matrix system, IHC studies revealed that the Gα_olf_ levels were normally found in both the striosome and matrix compartments in PD with LID, although they were markedly increased in the matrix compartment, but not or only mildly increased in the striosome compartment, in PD (Ruiz-DeDiego et al., 2015; Morigaki et al., 2017). This novel finding indicates that there is a difference in the dopaminergic regulation of the Gα_olf_ expression between the striosome and matrix compartments.

*In situ* proximity ligation assay (PLA) for dual-antigen recognition disclosed cell-type specific changes in the Gα_olf_ levels in the DA-depleted striatum of mice with and without LID (Morigaki et al., 2017). The* in situ* PLA technique can indicate the presence of the Gα_olf_ protein in close proximity to the D_1_R protein (D_1_R-Gα_olf_) or A_2A_R protein (A_2A_R-Gα_olf_). Quantitative *in situ* PLA showed that DA depletion caused a marked (~90%) increase in the striatal levels of D_1_R-Gα_olf_ PLA signals, which were downregulated by a daily administration of levodopa. However, there remained a significant (~50%) increase in the striatal D_1_R-Gα_olf_ PLA signals in mice with LID when compared with normal controls. On one hand, quantitative* in situ* PLA also disclosed that a daily exposure to levodopa, but not DA depletion *per se*, caused a significant (~40%) decrease in the striatal A_2A_R-Gα_olf_ PLA signals in the DA-depleted striatum of mice with LID. These findings indicate that, in the DA-depleted striatum, DA replacement could induce the downregulation of the Gα_olf_ protein levels not only in the striatonigral MSNs but also in the striatopallidal MSNs.

**Figure 3:**
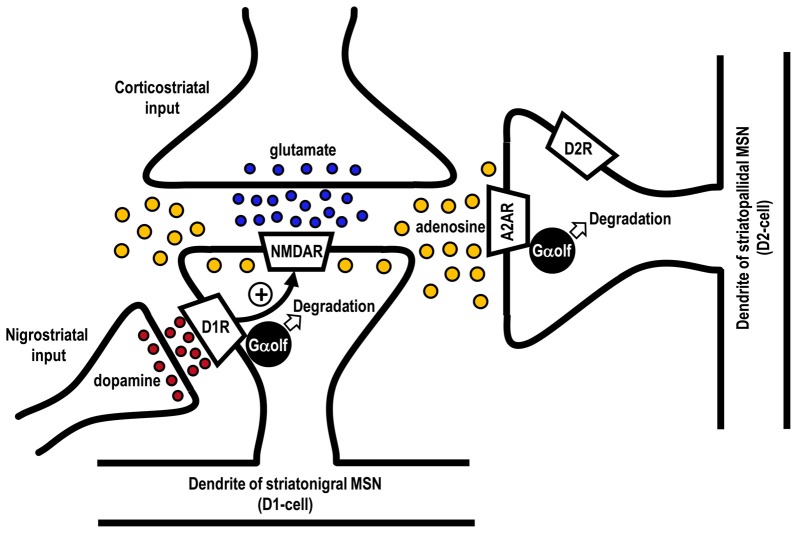
Possible mechanism for agonist-induced degradation of Gα_olf_ proteins in striatonigral and striatopallidal MSNs. Glutamate released from the corticostriatal afferents could activate postsynaptic *N-methyl-D-aspartate (NMDA)* receptors (NMDARs) to increase the extracellular adenosine levels in the striatum. Repeated exposure to levodopa might cause a pulsatile release of DA from the nigrostriatal afferents to activate DA D_1_ receptors (D_1_Rs) in striatonigral MSNs (D1-cells). This might facilitate the NMDAR-evoked increase in extracellular adenosine release and, thereby, indirectly activate the adenosine A_2A_ receptors (A_2A_Rs) in striatopallidal MSNs expressing DA D_2_ receptors (D_2_Rs; D2-cells). Thus, a usage-induced downregulation of Gα_olf_ protein levels could occur not only in the striatonigral MSNs but also in striatopallidal MSNs.

An intriguing question is how the Gα_olf_ protein levels are decreased in the striatopallidal MSNs in LID. In animal models with nigrostriatal 6-OHDA-lesions, persistent (chronic) DA depletion *per se* has been shown to cause no apparent changes (Ballarin et al., 1987; Herrera-Marschitz et al., 1994; Nomoto et al., 2000) or mild decrease (Pinna et al., 2002) in the extracellular levels of adenosine in the DA-denervated striatum. However, evidence shows that the striatal adenosine levels are elevated by the activation of NMDA receptors (Delaney and Geiger, 1998; Delaney et al., 1998), which can be enhanced by D_1_R activation (Cepeda and Levine, 2012; Morigaki and Goto, 2015; see Figure 3). Interestingly, a *pulsatile* exposure to the D_1_R agonist reportedly facilitated the NMDA receptor-evoked increase in the extracellular adenosine release in the rat striatum (Harvey and Lacey, 1997). This evidence suggests that, in the DA-depleted striatum with D_1_R hypersensitivity, a repeated administration of levodopa may exert a *pulsatile* activation of D_1_Rs, which subsequently facilitates the NMDA receptor-evoked increase in the extracellular adenosine levels. Moreover, in the DA-depleted striatum, the activation of NMDA receptor could lead to a marked increase in the extracellular adenosine levels and, then, indirectly activate A_2A_Rs (Nash and Brotchie, 2000). Thus, it is likely that the downregulation of the Gα_olf_ levels in striatopallidal MSNs in LID might result from an increased usage of Gα_olf_ proteins through the A_2A_R activation subsequent to the daily *pulsatile* activation of striatal D_1_Rs. This notion also suggests that the striatal D_1_R signals might play a critical role in the regulation of the Gα_olf_ protein levels not only in the striatonigral MSNs, but also in the striatopallidal MSNs in the DA-denervated striatum. This consideration may corroborate the general concept that increased activities of striatal D_1_Rs are requisite for the genesis of LID (Westin et al., 2007; Darmopil et al., 2009; Alcacer et al., 2012).

## Striatal Gα_olf_/cAMP Signal-Dependent Mechanism for Generating LID

**Figure 4:**
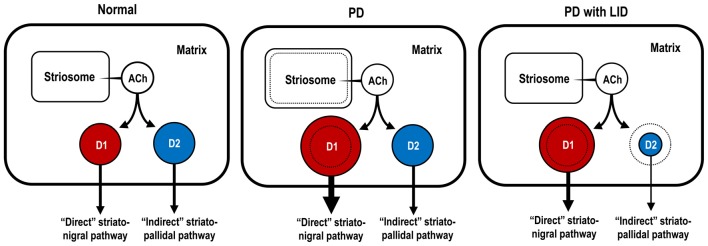
Hypothetical diagram for dopaminergic regulation of Gα_olf_ protein levels in striatonigral and striatopallidal MSNs. The sizes of the circles, colored in *red* and *blue*, indicate the abundance of Gα_olf_ proteins in striatonigral MSNs expressing dopamine D_1_ receptors (D_1_Rs; D1-cells; *red*) and in striatopallidal MSNs expressing dopamine D_2_ receptors (D_2_Rs; D2-cells; *blue*), respectively. In the conditions of Parkinson’s disease (PD), D1-cells, but not D2-cells, might exhibit a DA D_1_ hypersensitivity caused by a dramatic increase in their Gα_olf_ levels. In the conditions of PD with levodopa-induced dyskinesia (LID), D1-cells might show an increase in their Gα_olf_ levels, while D2-cells might show a decrease in their Gα_olf_ levels, which might result in an enhanced responsiveness to D_2_R activation. ACh, acetylcholine; D1-cell, striatonigral medium spiny neuron expressing DA D_1_ receptor; D2-cell, striatopallidal medium spiny neuron expressing DA D_2_ receptor; PD, Parkinson’s disease; PD with LID, Parkinson’s disease with levodopa-induced dyskinesia.

Figure 4 shows the hypothetical representation of the Gα_olf_ protein levels in striatonigral and striatopallidal MSNs in the DA-denervated striatum under the conditions of both PD with and without LID. In PD, there is a dramatic increase in the Gα_olf_ protein levels in the striatonigral MSNs, but not in the striatopallidal MSNs. Because of no apparent changes in the striatal D_1_R levels (Shinotoh et al., 1993; Turjanski et al., 1997; Hurley et al., 2001) and other principal mediators of the D_1_R signaling cascades (Girault et al., 1989; Nishino et al., 1993) in patients with PD, the marked increase in the Gα_olf_ protein levels in the striatonigral MSNs may be a principal cause for generating striatal D_1_R hypersensitivity to levodopa exposure in PD. This notion corroborates the evidence that there is a marked increase in the responsiveness of the striatonigral MSNs to D_1_R activation in PD, as determined by the fos induction experiments (Engber et al., 1989; Asin et al., 1995; Kashihara et al., 2000; Xu et al., 2003; Morigaki et al., 2017).

In PD with LID, there is an important decrease in the Gα_olf_ protein levels in the striatopallidal MSNs after a prolonged and pulsatile administration of levodopa. This leads to the facilitation of the effects of DA on striatopallidal MSNs by reducing the A_2A_R/Gα_olf_ signal-mediated cAMP production and subsequently to the increase in the responsiveness of striatopallidal MSNs to D_2_R activation. Indeed, it was importantly noted that, during the increasing phase of dyskinesias, an abnormal lowering of intracellular cAMP levels transiently occurred in the DA-denervated striatum in rat model of LID (Sancesario et al., 2014). These novel findings parallel the evidence that a repeated exposure to levodopa results in a significant increase in the responsiveness of striatopallidal MSNs to dopaminergic stimulation, as determined by fos induction experiments (Engber et al., 1989; Asin et al., 1995; Kashihara et al., 2000; Xu et al., 2003; Morigaki et al., 2017). In addition, there is a significant increase in the Gα_olf_ protein levels in striatonigral MSNs in PD with LID as compared to normal controls. Because Gα_olf_ is the regulator of cAMP signal-dependent activities in the striatum, an increase in the responsiveness of both striatonigral and striatopallidal MSNs to levodopa exposure, which depends on the Gα_olf_ protein levels, serves as a principal cause for generating LID.

## Concluding Remarks

Since the intracellular cAMP signaling cascades serve as a determinant of striatal cell activities (Girault, 2012), maladaptive change in Gα_olf_ protein levels is thought to be closely linked to the pathophysiology of PD (Hervé, 2011). Here, we hypothesized that DA depletion might cause a marked upregulation of the Gα_olf_ protein levels in striatonigral MSNs, which results in a crucial hypersensitivity of the striatum to D_1_R stimulation in PD. A *prolonged* and *pulsatile* exposure to levodopa might lead to a usage-dependent decrease in the Gα_olf_ protein levels not only in the nigrostriatal MSNs but also in the striatopallidal MSNs in PD with LID. This levodopa-induced decrease in Gα_olf_ protein levels, which might be due to a *pulsatile* activation of postsynaptic D_1_Rs and NMDA receptors, could result in reduced A_2A_R/Gα_olf_/cAMP signal levels in striatopallidal MSNs. This might cause an increase in the responsiveness of striatopallidal MSNs to D_2_R activation, and thereby develop LID in PD. Our hypothesis corroborates the long-lasting concept that LIDs are associated with a decreased activity of the “indirect” striatopallidal pathway (Crossman, 1990; DeLong, 1990; Brotchie, 2005).

As an important cellular mechanism to regulate the activities of striatal MSNs, the recurrent collateral connections between the MSNs have also been identified (Bolam et al., 1983; Yung et al., 1996). The activities of striatopallidal MSNs can be inhibited by the GABAergic collateral axon branches from neighboring MSNs (Taverna et al., 2008; Lalchandani et al., 2013; Dobbs et al., 2016; Wei et al., 2017). Thus striatal D_1_ hypersensitivity could lead to an increased responsiveness of striatopallidal MSNs to D_2_R activation in the conditions of PD with and without LID, although only a small population of the striatonigral MSNs has been found to form collateral axon connections with striatopallidal MSNs in the mouse striatum (Taverna et al., 2008). However, this notion *per se* could not explain the progressive increase in the severity of LID, which occurs in the PD patients treated with unaltered dosages of given dopaminergic drugs (Brotchie, 2005), because there is an ongoing decline in striatal responsiveness to D_1_R activation along a repeated exposure to levodopa under the conditions of PD, as determined by fos induction experiments (Saka et al., 1999; Kashihara et al., 2000; Xu et al., 2003; Morigaki et al., 2017).

Finally, we suggest that the pharmacological concomitant therapy to increase Gα_olf_ protein levels in the striatum might be useful in the management of LID and motor fluctuations in patients with PD treated with DA replacement therapy. The normalization of the decreased Gα_olf_ protein levels in the striatopallidal MSNs might suppress LID. On one hand, the elevation of the Gα_olf_ protein levels in the striatonigral MSNs could increase the striatal responsiveness to D_1_R activation and, thereby, facilitate the therapeutic efficacy of dopaminergic drugs. In considering the possible involvement of the activated NMDA receptors in lowering striatal Gα_olf_ levels in LID, NMDA receptor antagonists (e.g., amantadine or memantine) might attenuate LID, as already shown in clinical practice (Rascol et al., 2015). Because A_2A_R activation, which could reduce the Gα_olf_ protein levels in the striatopallidal MSNs leading to LID, might be required for the “priming” of LID (Brotchie, 2005; Xiao et al., 2006), it is suggested that A_2A_R antagonists (e.g., istradefylline) might be effective in dampening the “priming” of LID. However, after the establishment of LID, the adjunct use of A_2A_R antagonists might exacerbate the dyskinetic symptoms as shown in clinical practice (Kondo and Mizuno, 2015).

## Author Contributions

SG wrote the manuscript.

## Conflict of Interest Statement

The author declares that the research was conducted in the absence of any commercial or financial relationships that could be construed as a potential conflict of interest.
